# Community-Based Appraisal of the Effects of Parenteral Nutrition Versus Enteral Nutrition on the Quality of Care for Patients With Acute Pancreatitis

**DOI:** 10.4021/gr289w

**Published:** 2011-01-20

**Authors:** Kazuaki Kuwabara, Shinya Matsuda, Kiyohide Fushimi, Koichi B Ishikawa, Hiromasa Horiguchi, Kenji Fujimori

**Affiliations:** a Kyushu University Graduate School of Medical Sciences, Department of Health Care Administration and Management, 3-1-1 Maidashi, Higashi-ku, Fukuoka 812-8582, Japan; b University of Occupational and Environmental Health, 1-1 Iseigaoka Yahatanishi-ku Kitakyushu, Fukuoka 807-8555, Japan; c Tokyo Medical and Dental University, 1-5-45 Yushima Bunkyo-ku, Tokyo 113-8510, Japan; d National Cancer Center, 5-1-1 Tsukiji Chuo-ku, Tokyo 104-0045, Japan; e University of Tokyo Graduate School of Medicine, 7-3-1 Hongo Bunkyo-ku, Tokyo 113-0033, Japan; f Hokkaido University, 5 Nishi 14 Kita Kita-ku, Sapporo, Hokkaido 060-8648, Japan

**Keywords:** Enteral nutrition, Parenteral nutrition, Acute pancreatitis, Quality of care

## Abstract

**Background:**

Enteral nutrition (EN) rather than parenteral nutrition (PN) has been advocated in treatment guidelines for acute pancreatitis (AP) as endorsed in randomized studies or meta-analyses. The findings derived from those studies would recognize the criticism of smaller sample sizes or limited patient case-mixes. To determine the generalizability of those findings, community-based appraisal on the advantages of EN over PN is required. Using a Japanese administrative database between 2006 and 2010, we determine whether EN is superior to PN in the real clinical settings.

**Methods:**

A total of 24,913 patients diagnosed with AP at admission in 1,000 hospitals were identified. Among them, we analyzed 1,803 patients of ≥ 15 years who received EN or PN for AP across 480 hospitals. Among three nutrition categories of PN only, EN only and PN with EN, we examined patient characteristics, comorbidities, complications, AP severity score determined by the Ministry of Health, Labor and Welfare, surgical procedures for the biliary/pancreatic system, use of artificially assisted ventilation and hemodialysis proxy of multiple organ failures and hospital teaching status. To identify the variables associated with PN use, a logistic regression model was used and the propensity score (PS) was calculated to control for the selection bias of patient case-mix preferring PN use. Then, we compared mortality, length of hospital stay (LOS), total charges (TC) and commencement day of oral food intake between EN and PN.

**Results:**

A total of 1,191 PN patients, 330 EN patients and 282 mixed EN and PN patients were identified. EN was indicated for patients with mild AP and procedures for the pancreas. PS matching analysis indicated that PN had a higher mortality compared with EN, and PN significantly increased LOS and TC compared with EN. PN deterred the commencement of oral food intake.

**Conclusions:**

Community-based study has shown that EN was employed in the less severe case-mixed. Even though considering that selection bias, EN was still superior to PN in AP. Physicians should be aware of the guidelines for the advocacy of EN and need to carefully consider the indications for EN to optimize the quality of AP care.

## Introduction

Enteral nutrition (EN) has advantages over parenteral nutrition (PN), especially in patients with severe acute pancreatitis (AP), as shown by randomized studies and meta-analyses [1-8]. EN has been proven to be safer, is associated with a shorter length of hospital stay (LOS) and causes a decrease in the mortality rate [2, 3, 5-8].

Therefore, the implementation of EN is recommended in several sets of guidelines for AP management, and some guidelines recommend administering EN as soon as the patient can tolerate EN [2-6, 8].

However, these guidelines were mostly derived from randomized studies with limited patient case-mixes or meta-analyses of data from several types of studies with relatively small sample sizes [3, 4, 8]. These previous studies might be lacking in external validation, because each study method was heterogeneous and they often had a small sample size [3, 4, 8]. To the best of our knowledge, there have been few comprehensive community-based reappraisals on the advantages of EN over PN, using an administrative database. In some clinical settings, once EN is indicated, there might be some case-mixes that prevent the accomplishment of EN care, such as surgical procedures on the pancreatic system, critical care administration representing persistent multiple organ failure (MOF), intolerance of EN hindering sufficient calorie intake, and/or pneumonia from aspiration of a liquid supplement.

Using a Japanese administrative database containing patient case reports, we examined whether EN was better than PN in terms of mortality, LOS, medical expenditures and when oral intake of semi-solid food commenced.

## Materials and Methods

We carried out a 6-month observational study using a Japanese administrative database with data received annually from July 1 to December 31 between 2006 and 2010. This database was originally established by the Japanese Ministry of Health, Labor and Welfare (MHLW) and our research team, and it consists of discharge summaries and anonymous health insurance claim data. It has been used in cooperation with our research project and several clinical societies to develop a Japanese case-mix classification and to assess hospital performance and payments in 1,607 hospitals in 2010. These hospitals provide acute care, promote medical research and train medical students and postgraduate specialty trainees. From a total of 24,913 patients diagnosed with AP at admission in 1,000 hospitals, we identified 1,803 patients aged ≥ 15 years who had available data for the AP severity score determined by the MHLW (JPN score) and they received either EN or PN in 480 hospitals that participated in our research project. The study was approved by the ethical committee of the University of Occupational and Environmental Health, Fukuoka, Japan.

### Variable definitions

We studied three nutritional support groups: PN, EN, and a combination of PN and EN (EN consisted of semi-elemental formula or polymeric formula). PN in this study excluded fat emulsion. We compared the following variables: age, sex, use of an ambulance, JPN score, pre-existing comorbidities, complications, hospital teaching status (community or academic hospitals including university hospitals and the National Cancer Center and National Cardiovascular Center), care process including protease inhibitors (aprotinin, camostat mesilate, gabexate mesilate, nafamostat mesilate, octreotide, and ulinastatin), use of artificially assisted ventilation and hemodialysis, surgical procedures on the biliary system or pancreas (cholecystectomy, choledocholithotomy, endoscopic sphincterotomy or balloon dilatation of the papilla of Vater, endoscopic stent insertion into the common bile duct, transhepatic biliary drainage, necrosectomy, pancreatic resection, and drainage of abscesses or of pancreatic cysts by cystogastrostomy/enterostomy), and the day when oral intake of semi-solid food commenced. LOS and total charges (TC; 1 euro = 120 yen) were calculated. TC correlates well with in-hospital costs [9].

Diagnoses were coded according to the International Classification of Disease 10th version (ICD-10), and study patients were those who had an ICD-10 code of K85 indicating AP at admission. Patients were stratified into two age groups: < 65 years and ≥ 65 years. The JPN score was determined by clinical signs and laboratory data. Two points were assigned for each of the following nine factors: shock, respiratory failure, mental disturbance, severe infection, hemorrhagic diathesis, base excess ≤ 3 mEq/l, hematocrit ≤ 30% after hydration, blood urea nitrogen ≥ 40 mg/dl or creatinine ≥ 2 mg/dl, and a systemic inflammatory response score (SIRS) ≥ 3. [The SIRS was determined by a number of the following criteria: heart rate > 90 beats/minute, respiratory rate > 20 breaths/minute, body temperature > 38 °C or < 36 °C, leukocyte blood count > 12 x 10^3^/mm^3^ or < 4 x 10^3^/mm^3^]; 1 point was assigned for the following nine factors: age ≥ 70 years, Ca ≤ 7.5 mg/dl, fasting blood sugar ≥ 200 mg/dl, PaO_2_ ≤ 60 mmHg in room air, lactate dehydrogenase ≥ 700 IU/L, total protein ≤ 6.0 g/dl, prothrombin time ≥ 15 seconds, platelet count ≤ 10^5^/mm^3^ and a computed tomography grade of 4 or 5. Severity of AP was classified as mild AP (0 points), moderate AP (1 point), severe AP 1 (2 - 8 points), severe AP 2 (9 - 14 points) and extremely severe AP (15 - 27 points) [10]. In addition to this score, we examined ventilation and hemodialysis representing persistent MOF in this study.

The database recorded a maximum of either four pre-existing comorbidities or four complications during hospitalization per patient. The Charlson comorbidity index (CCI) was used to assess the severity of pre-existing co-morbid conditions [11]. Patients were divided into five groups according to CCI: 0, 1, 2, 3 or ≥ 4. Study complications included procedure-related complications such as wound complications, catheter-related infection and others (T81 - T87), mechanical bowel obstruction (K565-7, K913) and recurring acute pancreatitis (K85) or peritonitis/intra-abdominal abscess [12]. EN administration was divided into two groups: (1) EN was performed within 2 days after admission and (2) EN was performed longer than 2 days after admission.

### Statistical analysis

Frequencies and percentages for categorical data in the three groups were compared by the Pearson chi-test and continuous variables were compared using analysis of variance. To identify the variables associated with PN, a logistic regression model was used and the propensity score (PS) was calculated to control for the selection bias of patient case-mix for PN use [13]. We constructed PS matching cohorts and used logistic regression to measure the impact of PN on mortality. We also used a mixed linear regression model where individual hospital was handled as random intercept to standardize variations in hospital practices to estimate the impact of PN on LOS, TC and the day that oral food intake was initiated. Statistical analysis was performed using SPSS version 16.0, with the level of significance set at P < 0.05.

## Results

There were 1,191 PN patients in 423 hospitals, 330 EN patients in 156 hospitals, and 282 EN plus PN patients in 146 hospitals. The mean age did not differ significantly between nutritional support categories, but the percentage of ambulances used, severity of the JPN score, and the use of protease inhibitors were significantly less in EN patients than those in PN patients (Table 1).

**Table 1:** Patient Characteristics, Care Process and Resource Use Among Study Nutritional Approach (%)

			PN only	EN only	PN + EN	P
Overall			1191	330	282 (242^a^)	
Number of hospitals (community, academic)			370, 53	135, 21	116, 30	
Age	Mean, [SD]		60.8 [17.3]	58.5 [19.0]	58.9 [16.6]	0.366^†^
	40 - 64 years		495 (41.6)	129 (39.1)	115 (40.8)	0.263
	≥ 65 years		531 (44.6)	140 (42.4)	119 (42.2)	
Gender						
	Male		793 (66.6)	229 (69.4)	208 (73.8)	0.059
Ambulance						
	Used		426 (35.8)	104 (31.5)	122 (43.3)	0.009
Outcome						
	Mortality		94 (7.9)	6 (1.8)	28 (9.9)	< 0.001
JPN score						
	Mild AP		225 (18.9)	103 (31.2)	29 (10.3)	< 0.001
	Moderate AP		279 (23.4)	77 (23.3)	35 (12.4)	
	Severe AP 1		564 (47.4)	130 (39.4)	152 (53.9)	
	Severe AP 2		85 (7.1)	14 (4.2)	46 (16.3)	
	Extremely severe AP		38 (3.2)	6 (1.8)	20 (7.1)	
Charlson comorbidity index						
	1		290 (24.3)	80 (24.2)	70 (24.8)	0.141
	2		88 (7.4)	29 (8.8)	37 (13.1)	
	3		42 (3.5)	8 (2.4)	8 (2.8)	
	≥ 4		13 (1.1)	6 (1.8)	3 (1.1)	
Complication						
	Overall		65 (5.5)	6 (1.8)	26 (9.2)	0.009
	Recurrent AP		12 (1.0)	1 (0.3)	8 (2.8)	
	Bowel obstruction		26 (2.2)	3 (0.9)	9 (3.2)	
	Relapsing peritonitis/intraabdominal abcess		17 (1.4)	1 (0.3)	5 (1.8)	
Hospital category						
	Academic		196 (16.5)	51 (15.5)	63 (22.3)	0.008
Protease inhibitor						
	Overall		1173 (98.5)	318 (96.4)	280 (99.3)	0.012
	Camostat mesilate		527 (44.2)	156 (47.3)	138 (48.9)	
	Gabexate mesilate		903 (75.8)	170 (51.5)	191 (67.7)	
	Nafamostat mesilate		534 (44.8)	167 (50.6)	188 (66.7)	
	Octreotide acetate		44 (3.7)	12 (3.6)	24 (8.5)	
	Ulinastatin		605 (50.8)	162 (49.1)	185 (65.6)	
Enteral nutrition						
	Use within 2 days		0 (0.0)	14 (4.2)	12 (4.3)	
	Use after 3 days		0 (0.0)	316 (95.8)	270 (95.7)	
Ventilation						
	Present		90 (7.6)	11 (3.3)	52 (18.4)	< 0.001
Hemodialysis						
	Present		102 (8.6)	17 (5.2)	47 (16.7)	< 0.001
Surgical procedures for the biliary tract						
	Present		217 (18.2)	53 (16.1)	58 (20.6)	0.354
Surgical procedures for the pancreas						
	Present		51 (4.3)	1 (0.3)	28 (9.9)	< 0.001
Oral food intake						
	Present		1095 (91.9)	317 (96.1)	264 (93.6)	0.031
Commencement day of oral food intake (days)						
	Mean days [SD]		16.4 [14.0]	10.9 [8.3]	21.2 [17.2]	< 0.001^†^
Length of hospital stay (days)						
	Mean [SD]		36.5 [25.7]	26.8 [19.6]	56.9 [34.3]	< 0.001^†^
Total charge (Euros)						
	Mean [SD]		13,725 [12,360]	9,755 [10,962]	27,316 [23,363]	< 0.001^†^

PN, parenreral nutrition; EN, enetral nutrition; a, cases of preceding TPN; SD, standard deviation; AP, acute pancreatitis; JPN score, the severity scoring system determined by the Ministry of Health and Welfare of Japan.

†: Compared by analysis of variance. Other variables were compared by chi-square test.

The severity of JPN scores and surgical procedures for the pancreas were associated with less employment of EN (Table 2).

**Table 2:** Variables Associated With Indication of Enteral Nutrition

		Odds ratio	[95% CI]
Age (for 15 - 40 years)			
	40 - 64 years	0.750	[0.528 - 1.065]
	≥ 65 years	0.806	[0.564 - 1.152]
Gender			
	Male	1.113	[0.846 - 1.465]
Ambulance			
	Used	0.909	[0.697 - 1.184]
JPN score (for mild AP)			
	Moderate AP	0.605	[0.430 - 0.852]
	Severe AP 1	0.446	[0.330 - 0.604]
	Severe AP 2	0.273	[0.149 - 0.501]
	Extreme severe AP	0.281	[0.116 - 0.680]
Charlson comorbidity index (for zero)			
	1	0.985	[0.736 - 1.318]
	2	1.098	[0.704 - 1.714]
	3	0.689	[0.318 - 1.493]
	≥ 4	1.782	[0.669 - 4.743]
Surgical procedures for the biliary tract			
	Present	0.234	[0.008 - 6.595]
Surgical procedures for the pancreas			
	Present	0.059	[0.008 - 0.430]
Teaching status (for community)			
	Academic	0.889	[0.634 - 1.246]
Hosmer Lemeshow goodness of model fit		0.202	

CI, confidence interval; AP, acute pancreatitis; JPN score, the severity scoring system determined by the Ministry of Health and Welfare of Japan.

Using propensity score matching analysis, 370 patients were allocated into the PN group (298 PN alone and 72 patients had earlier commenced PN than EN) and the EN group (330 EN alone and 40 patients had preceding EN administration). There were fewer complications, a decreased mortality, less frequent requirements for ventilation and hemodialysis, an earlier return to oral food intake, a shorter LOS and less TC in EN patients compared with PN alone patients or PN plus EN patients (Table 3).

**Table 3:** Patient Characteristics, Care Process and Resource Use Among Study Nutritional Approach on the Propensity Score Matching Cohorts (%)

		PN only	EN only	PN + EN	P
Overall		298	330	112 (72^a^)	
Number of hospitals (community, academic)		147, 15	135, 21	48, 15	
Age	Mean, [SD]	59.4 [17.8]	58.5 [19.0]	56.6 [16.7]	0.001^†^
	40 - 64 years	125 (41.9)	129 (39.1)	50 (44.6)	0.592
	≥ 65 years	124 (41.6)	140 (42.4)	39 (34.8)	
Gender					
	Male	201 (67.4)	229 (69.4)	86 (76.8)	0.183
Ambulance					
	Used	83 (27.9)	104 (31.5)	49 (43.8)	0.009
Outcome					
	Mortality	18 (6.0)	6 (1.8)	12 (10.7)	< 0.001
JPN score					
	Mild AP	91 (30.5)	103 (31.2)	16 (14.3)	< 0.001
	Moderate AP	77 (25.8)	77 (23.3)	15 (13.4)	
	Severe AP 1	110 (36.9)	130 (39.4)	63 (56.3)	
	Severe AP 2	16 (5.4)	14 (4.2)	14 (12.5)	
	Extremely severe AP	4 (1.3)	6 (1.8)	4 (3.6)	
Charlson comorbidity index					
	1	86 (28.9)	80 (24.2)	35 (31.3)	0.394
	2	22 (7.4)	29 (8.8)	15 (13.4)	
	3	7 (2.3)	8 (2.4)	4 (3.6)	
	≥ 4	7 (2.3)	6 (1.8)	2 (1.8)	
Complication					
	Overall	13 (4.4)	6 (1.8)	9 (8.0)	< 0.001
	Recurrent AP	3 (1.0)	1 (0.3)	3 (2.7)	
	Bowel obstruction	6 (2.0)	3 (0.9)	3 (2.7)	
	Relapsing peritonitis/intraabdominal abcess	3 (1.0)	1 (0.3)	4 (3.6)	
Hospital category					
	Academic	27 (9.1)	51 (15.5)	22 (19.6)	< 0.001
Protease inhibitor					
	Overall	290 (97.3)	318 (96.4)	111 (99.1)	0.313
	Camostat mesilate	136 (45.6)	156 (47.3)	57 (50.9)	
	Gabexate mesilate	219 (73.5)	170 (51.5)	71 (63.4)	
	Nafamostat mesilate	130 (43.6)	167 (50.6)	81 (72.3)	
	Octreotide acetate	4 (1.3)	12 (3.6)	7 (6.3)	
	Ulinastatin	146 (49.0)	162 (49.1)	72 (64.3)	
Enteral nutrition					
	Use within 2 days	0 (0.0)	14 (4.2)	10 (8.9)	
	Use after 3 days	0 (0.0)	316 (95.8)	102 (91.1)	
Ventilation					
	Present	15 (5.0)	11 (3.3)	16 (14.3)	< 0.001
Hemodialysis					
	Present	22 (7.4)	17 (5.2)	19 (17.0)	< 0.001
Surgical procedures for the biliary tract					
	Present	54 (18.1)	53 (16.1)	19 (17.0)	0.790
Surgical procedures for the pancreas					
	Present	4 (1.3)	1 (0.3)	7 (6.3)	< 0.001
Oral food intake					
		280 (94.0)	317 (96.1)	107 (95.5)	0.463
Commencement day of oral food intake (days)					
	Mean days [SD]	14.5 [9.7]	10.9 [8.3]	18.5 [17.9]	< 0.001^†^
Length of hospital stay (days)					
	Mean [SD]	36.0 [24.4]	26.8 [19.6]	53.6 [36.8]	< 0.001^†^
Total charge (Euros)					
	Mean [SD]	12,298 [8,510]	9,755 [10,962]	25,311 [20,158]	< 0.001^†^

PN, parenreral nutrition; EN, enetral nutrition; a, cases of preceding TPN; SD, standard deviation; AP, acute pancreatitis; JPN score, the severity scoring system determined by the Ministry of Health and Welfare of Japan.

†: Compared by analysis of variance. Other variables were compared by chi-square test.

The use of PN was significantly correlated with an increased mortality (odds ratio, 2.353; 95% confidence interval (CI), [1.051 - 5.268]), a longer LOS (days) (unstandardized coefficient, 7.5; 95% CI, [4.0 - 11.1]), higher TC (Euros) (2400, CI [902 - 3897]) and delayed commencement of oral intake (days) (2.3, [0.7 - 3.9]). Hemodialysis was correlated with a high mortality. Surgical procedures for the pancreas and biliary tract significantly increased LOS and TC and delayed the commencement of oral food intake. Ventilation and hemodialysis were significantly associated with TC but not with the day of return to oral food intake (Table 4).

**Table 4:** Variables Associated With Mortality, Resource Use and Commencement Day of Oral Food Intake

		Mortality		Length of hospital stay (days)		Total charge (Euros)		Commencement day of oral food intake (days)	
		Odds ratio	[95% CI]	Estimation	[95% CI]	Estimation	[95% CI]	Estimation	[95% CI]
Intercept				16.0	[4.1 - 27.9]	2,278	[-2,762 - 7,319]	7.5	[2.4 - 12.7]
Age (for 15 - 40 years)									
	40 - 64 years	1.048	[0.278 - 3.950]	4.8	[-0.3 - 9.8]	-602	[-2,746 - 1,543]	0.2	[-2.1 - 2.4]
	65 - years	3.240	[0.905 - 11.601]	1.5	[-3.4 - 6.3]	-58	[-2,106 - 1,990]	1.4	[-0.8 - 3.5]
Gender									
	Male	1.800	[0.727 - 4.457]	-3.9	[-7.8 - 0.0]	-856	[-2,493 - 782]	-0.6	[-2.3 - 1.1]
Ambulance									
	Used	1.422	[0.647 - 3.128]	-1.9	[-5.7 - 2.0]	413	[-1,212 - 2,038]	-2.0	[-3.7 - -0.3]
JPN score (for mild AP)									
	Moderate AP	0.452	[0.121 - 1.687]	-0.2	[-5.0 - 4.6]	-321	[-2,355 - 1,713]	1.5	[-0.7 - 3.6]
	Severe AP 1	0.885	[0.334 - 2.343]	3.0	[-1.3 - 7.4]	2,130	[285 - 3,975]	2.1	[0.2 - 4.1]
	Severe AP 2	2.162	[0.590 - 7.927]	10.3	[2.4 - 18.3]	8,209	[4,847 - 11,571]	6.1	[2.5 - 9.7]
	Extremely severe AP	1.654	[0.281 - 9.750]	18.0	[4.9 - 31.1]	14,931	[9,400 - 20,461]	9.3	[3.2 - 15.4]
Charlson comorbidity index (for zero)									
	1	1.047	[0.290 - 16.624]	3.1	[-0.8 - 7.1]	771	[-901 - 2,442]	-0.5	[-2.3 - 1.3]
	2	2.411	[0.393 - 12.869]	4.2	[-2.0 - 10.4]	2,475	[-140 - 5,090]	-1.0	[-3.7 - 1.7]
	3	2.248	[0.836 - 6.952]	-0.3	[-11.1 - 10.6]	-880	[-5,465 - 3,704]	-2.0	[-6.7 - 2.7]
	≥ 4	2.195	[0.412 - 2.659]	4.2	[-8.2 - 16.6]	807	[-4,424 - 6,038]	-0.4	[-6.0 - 5.2]
Surgical procedures for the biliary tract									
	Present	2.348	[0.988 - 5.581]	9.3	[4.6 - 13.9]	5,073	[3,113 - 7,034]	2.1	[0.0 - 4.2]
Surgical procedures for the pancreas									
	Present	0.570	[0.051 - 6.357]	48.0	[33.9 - 62.1]	19,740	[13,785 - 25,694]	28.9	[22.4 - 35.3]
Protease inhibitor									
	Present	**		8.7	[-2.0 - 19.4]	5,149	[639 - 9,659]	2.8	[-1.8 - 7.4]
PN									
	Present	2.353	[1.051 - 5.268]	7.5	[4.0 - 11.1]	2,400	[902 - 3,897]	2.3	[0.7 - 3.9]
Ventilation									
	Present	2.240	[0.832 - 6.030]	1.9	[-6.4 - 10.3]	12,522	[8,991 - 16,053]	1.5	[-2.2 - 5.2]
Hemodialysis									
	Present	7.979	[2.615 - 24.350]	16.6	[9.6 - 23.7]	14,666	[11,702 - 17,630]	2.5	[-0.6 - 5.7]
Complication									
	Present	0.929	[0.173 - 4.984]	15.0	[5.9 - 24.0]	5,924	[2,106 - 9,742]	-0.1	[-4.2 - 4.0]
Teaching status (for community)									
	Academic	0.899	[0.291 - 2.780]	-1.2	[-6.9 - 4.6]	1,210	[-1,206 - 3,626]	0.4	[-2.1 - 2.9]
Goodness of model fit		0.667^a^		6809.3^b^		15757.2^b^		5304.8^b^	

^**^protease was absent in cases that survived. CI, confidence interval; PN, parenreral nutrition; AP, acute pancreatitis; JPN score, the severity scoring system determined by the Ministry of Health and Welfare of Japan. Model fitness was measured in “a” by Hosmer Lemeshow, and in “b” by Akaike information criteria.

## Discussion

We used a Japanese administrative database to compare the advantages of EN over PN in terms of mortality, LOS, TC, and the commencement of oral semi-solid food intake as determined by propensity score matching analysis. EN was indicated less frequently in severe AP cases, but overall, EN was associated with a better outcome than PN in AP patients.

Previous studies on the advantages of using EN over PN have included randomized studies with a small patient sample size or meta-analyses. To the best of our knowledge, this is the first community-based appraisal on the advantages of EN over PN using a large sample size and providing detailed information about the care process. Our study attempted to eliminate the variability in severity scores and discrepancies in the definitions of MOF or formulation of PN, as these parameters were not taken into account in previous meta-analyses [4]. The JPN score includes laboratory tests and clinical findings and has a similar value as the APACHE score and Ranson score [10]. However, these values do not always represent persistent MOF. In addition, there has been criticism that physiological severity scores such as the American Society of Anesthesiologists might be subjective. A more objective indicator such as ventilation or hemodialysis that might be more representative of MOF may be more practical and feasible in this administrative database [14]. Ventilation and hemodialysis (indicating persistent MOF) were used in this study.

Our findings are similar to those in several meta-analysis reports in which data from randomized clinical studies were collected [3, 6, 8]. Most of the previous studies assessed the impact of EN or PN on the use of ventilation administration and surgery in addition to other parameters such as complications, mortality and LOS. To evaluate the real cost during hospitalization, the effects of surgical procedures or critical care should not be underestimated. In the current study, surgical procedures for the biliary tract or pancreas, ventilation and hemodialysis were significant determinants of healthcare cost. Several randomized studies using meta-analyses had very small patient sample sizes and they did not collect information about surgical procedures [2, 3, 5, 6]. Surgical procedures for the pancreas are rarely performed, as indicated by our finding that only 5.0% of severe to extremely severe AP cases received surgical procedures. Our study found that overall LOS of AP patients appeared to be longer than that in Western studies; LOS in Japan is usually two to three times longer than that in Western countries [15]. Japanese hospitals generally supply wound care and nursing home services to reduce the burden of the patient and their family, in addition to acute medical care [16]. These longer admissions could considerably count towards the total cost for AP management.

Among various studies, there is heterogeneity in patient demographics, inclusion criteria, severity of AP, and outcomes [4, 5]. Variation in the patient-mix might influence the accomplishment of EN during hospitalization in the clinical setting. Therefore, community-based appraisal of the advantages of EN over PN could help establish AP guidelines, as well as improve the generalizability of the evidence obtained from different randomized studies.

PN is frequently employed in severe AP, which is a condition that does not permit oral intake or EN. To comprehensively evaluate the advantages of EN over PN by a community-based study approach, various factors need to be taken into account such as a selection bias that favors the use of PN; mechanical bowel obstruction, surgical procedures and critical care are all important factors in the choice for nutritional support for patients with severe AP. Combined PN and EN are recommended when adequate nutrition given by EN alone cannot be tolerated by patients [7, 17]. In the current study, we comprehensively evaluated several variables affecting the type of nutritional delivery to patients or resource use. The amount or percentage of caloric intake from PN and/or EN, as well as other nutrient formulations, such as immune enhanced formulation or prebiotics, influence patient outcome and resource use. Our administrative database contains data concerning such relevant details including the number of calories per unit, which could provide help in determining the efficacy of the different types of nutritional support in future studies.

There are several limitations to this study. First, the study period was limited to only 6 months, which could have diminished the generalizability of our results. However, the MHLW have extended the study period to 12 months starting in 2010. Second, clinical information such as body mass index or the actual EN route via nasogastric or nasojejunal feeding were not entered into the database [18]. The MHLW will also begin to collect patient height and body weight in 2010, and our administrative database includes the use of specific types of tubes such as nasogastric tubes, or a long tube extending beyond the ligament of Treitz into the duodenum. Since this administrative database contained the date and the quantity of every medical care item, we were able to determine the sequence of relevant care procedures and the causality among the care procedures, and subsequent outcomes could be clarified.

In conclusion, this community-based study demonstrated that PN is employed in severe AP, and that EN is safer and offers a more cost-efficient alternative for nutritional support, as well as promotes an earlier return to oral food intake compared with PN. Physicians should be aware of the guidelines advocating EN and carefully consider the indications for EN to optimize the quality of AP care. Future studies are required to evaluate in more detail the nutritional and caloric composition of EN and PN formulations, as well as to determine whether optimal timing for commencement of EN would contribute to a reduction in mortality, complications, requirement for critical care, and spare overall hospital resource use.
